# Childhood Arthritis and Rheumatology Research for Dreams Come True Remission

**DOI:** 10.3390/children9030324

**Published:** 2022-03-01

**Authors:** Syuji Takei

**Affiliations:** Department of Pediatrics, Field of Developmental Medicine, Graduate School of Medical and Dental Sciences, Kagoshima University, Kagoshima 890-8520, Japan; syujitakei@gmail.com

**Keywords:** pediatric rheumatic diseases, juvenile idiopathic arthritis (JIA), childhood-onset systemic lupus erythematosus (cSLE), dreams come true remission, new treatment goal, adolescent, transition

## Abstract

In recent years, newly developed therapeutic agents have brought clinical, structural, and functional remission to many pediatric patients with rheumatic diseases that were refractory to conventional therapy. However, achieving these remissions alone is insufficient as a treatment goal, especially for adolescent patients, because advanced therapies have not always encouraged their psychosocial stability and mental maturity. Consequently, various problems have arisen during the puberty and transition period from pediatrics to adult medical care. “Dreams come true remission” is a state of remission that allows patients to have clear dreams for the future in childhood and to increase the potential that their dreams will be realized in adulthood. This new treatment goal may empower children with chronic diseases such as PRDs to overcome the problems occurred during puberty and transition period.

Pediatric rheumatic diseases (PRDs) are a diverse group of inflammatory diseases that occurs in children below 18 years of age. The major PRDs are juvenile idiopathic arthritis (JIA), childhood-onset systemic lupus erythematosus (cSLE), and juvenile dermatomyositis (JDM). Most PRDs are chronic and intractable, often causing severe and permanent damage to the musculoskeletal system and organ functions in growing children.

In recent years, however, the prognosis of rheumatic diseases has improved dramatically with newly developed medications, such as monoclonal antibodies against inflammatory cytokines and Janus kinase (JAK) inhibitors, that can control inflammation and abnormal immune pathology in rheumatic diseases. These drugs are called biologic disease-modifying anti-rheumatic drugs (bDMARDs) and targeted synthetic DMARDs (tsDMARDs), respectively, and can modify the disease condition and induce clinical remission. Some of these DMARDs have been gradually introduced to pediatric patients. Consequently, patients with PRDs who were refractory to conventional therapies were brought into remission, and their clinical and functional (joint and organ) prognoses improved significantly.

These therapeutic advances have had a profound impact on the treatment strategies of adult patients with rheumatic diseases, and more objective and well-defined treatment strategies have been formulated for rheumatoid arthritis (RA) [1] and adult SLE [2]. In a novel treatment principle called treat-to-target (T2T), objective clinical assessment values are set as treatment targets. The therapeutic concepts of T2T have already been used for RA and adult SLE. The ultimate treatment goal is to achieve and maintain three types of remission: clinical, structural (imaging remission), and functional remission, with numerical goals for each remission. However, achieving these remissions alone is insufficient as a treatment goal for children with rheumatic diseases.

Physical growth and development are essential characteristics of children. However, persistent inflammation observed in rheumatic diseases stunts the physical characteristics of children with PRDs. In addition, persistent pain and limitations in daily life due to the disease negatively impact the psychological well-being of children with the disease, thus impeding their mental development. Especially in adolescent patients, the psychosocial impact of PRDs has a serious impact on the process of establishing oneself and becoming independent.

Therefore, ensuring physical growth and development, as well as psychosocial stability and healthy mental maturation, is an important therapeutic goal for patients with PRDs. In line with this, newer agents such as bDMARDs have been able to bring many PRD patients into remission, ensuring not only clinical, structural, and functional remission, but also physical growth. However, ensuring psychosocial stability and healthy mental maturation remains the most difficult therapeutic goal, especially in adolescents.

Dreams come true remission, discussed in this Special Issue, is a state of remission that allows children with rheumatic diseases to have clear dreams and hopes for the future in childhood and to increase the potential that these dreams will be realized in adulthood [3]. Achieving and maintaining this ‘dreams come true remission’ may lead to psychosocial stability and healthy mental maturation, especially in adolescents.

Puberty is a difficult time not only for sick children, but also for healthy children. It is often described as a period of confusion before the establishment of the ego and independence. Further, puberty represents a constant struggle between self-awareness and reality and is always dominated by ambivalence. Therefore, many adolescents are unable to foresee their future and are plagued by vague anxiety. In adolescents with PRDs, this condition is compounded by anxiety about the future caused by the disease, specifically regarding disease progression, employment and career choices, marriage, and pregnancy. In addition, adolescence is a stage of neurophysiological maturation in the limbic system and the frontal cortex, including myelination of nerve fibers, synaptic proliferation and pruning [4].

Therefore, without a clear goal for the future, it is difficult for children with PRDs to overcome this unstable and difficult period.

During the adolescent period, various human relations and life choices exist. Adolescents have to deal with peer relationships, romantic love, going to college or vocational school, and later finding a job. Simultaneously, most patients with PRDs transfer from pediatrics to adult rheumatology departments, and their medical environment changes dramatically. Consequently, various medical problems arise during the transition period.

The most common problem that occurs during the transition period is reduced compliance with medical visits. Bitencourt et al. studied 141 pediatric patients with rheumatic diseases who were transferred from pediatrics to the adult department [5], and reported that five (4%) died and 8 (6%) developed end-stage renal failure during a three-year transfer period, and all of them were cSLE. They also reported that after transferring to the adult rheumatology department, many patients were not seen on the day of their appointments and ended up in the emergency room or emergency hospitalization. These problems caused by the reduced compliance with medical visits can be avoided if patients have definite dreams or goals for their future by the time they are transferred to adult medical care.

On the other hand, there are many patients with PRDs who have realized their dreams in adulthood. In a report on the employment status of PRD patients in adulthood [6], the percentage of patients working full-time (42.1%) was lower than that of the general population of the same age (60.9%) in Japan, but the percentage of patients working in the medical profession (39.7%) was three times higher than that of the general population of the same age (14.8%). In the pilot study that preceded this survey [3], 42 (38.2%) of the 110 PRD patients who responded to the survey were employed in the medical profession, including 11 medical doctors, 17 nurses, 2 dentists, and 3 medical social workers.

These results may indicate that this is the one of the outcomes of ‘dreams come true remission’. The studies were conducted at facilities specializing in pediatric rheumatology. Patients with PRDs may have been closely exposed to medicine from an early age through the medical care of pediatric rheumatologists and aspired to become medical professionals in the future.

To achieve “dreams come true remission” in the clinical practice for patients with PRDs, pediatric rheumatologists must not only provide specialized medical care aimed at persistent clinical, structural, and functional remission, but also draw out and encourage their dreams for the future.

Similarly, we hold an annual meeting for patients with PRDs and their families, which include a peer counseling session for the future of children with PRD. Adults with PRDs who had transferred to the adult rheumatology department are asked to participate in this meeting as volunteers and to share their own experiences with children with PRDs and their families. This is peer counseling for the future of PRD children. In fact, there have been cases of children with JIA who later became medical doctors after being inspired by the experiences of an adult patient with JIA who became a physician.

I hope that this Special Issue will help all readers who work for chronically ill children to recognize the importance of achieving and maintaining the remission that allows these patients to have dreams for their future and to make their dreams really come true in adulthood.
